# RNA‐methyltransferase Nsun5 controls the maternal‐to‐zygotic transition by regulating maternal mRNA stability

**DOI:** 10.1002/ctm2.1137

**Published:** 2022-12-10

**Authors:** Chenyue Ding, Jiafeng Lu, Jincheng Li, Xiujuan Hu, Zhenxing Liu, Han Su, Hong Li, Boxian Huang

**Affiliations:** ^1^ State Key Laboratory of Reproductive Medicine Suzhou Affiliated Hospital of Nanjing Medical University Suzhou Municipal Hospital Gusu School Nanjing Medical University Suzhou China

## Abstract

**Background:**

RNA modification‐induced ovarian dysgenesis appears to be necessary for ovary development. However, how m^5^C (5‐methylcytosine)‐coordinating modificatory transcripts are dynamically regulated during oogenesis, and ovarian development is unknown. The purpose of this study was to determine whether NOP2/Sun RNA methyltransferase 5 (Nsun5) deletion leads to suppression of ovarian function and arrest of embryonic development. The regulation of mRNA decay and stability by m^5^C modification is essential at multiple stages during the maternal‐to‐zygotic (MZT) transition.

**Methods:**

Mouse ovaries and oocytes with *Nsun5* ^KO^ and the KGN cell line were subjected to m^5^C identification, alternative splicing analysis and protein expression. BS‐m^5^C‐seq, real‐time polymerase chain reaction, Western blot, immunofluorescence and actinomycin D treatment assays were used. In particular, BS‐m^5^C‐seq revealed a dynamic pattern of m^5^C sites and genes in the ovaries between *Nsun5* ^KO^ and WT mice at the 2‐month and 6‐month stages. Diverse bioinformatic tools were employed to identify target genes for Nsun5.

**Results:**

Here, a maternal mRNA stability study showed that deletion of the m^5^C methyltransferase Nsun5 obstructs follicular development and ovarian function, which leads directly to inhibition of embryogenesis and embryo development. Dynamic analysis of m^5^C revealed that the level of m^5^C decreased in a time‐dependent manner after Nsun5 knockout. Regarding the molecular mechanism, we found that Nsun5 deficiency caused a m^5^C decline in the exon and 3′UTR regions that influenced the translation efficiency of Mitotic arrest deficient 2 like 2 (MAD2L2) and Growth differentiation factor 9 (GDF9) in the ovary. Mechanistic investigation of alternative splicing indicated that *Nsun5* ^KO^ triggers aberrant events in the exon region of *Brd8*.

**Conclusions:**

Nsun5 loss arrests follicular genesis and development in ovarian aging, indicating that Nsun5/m^5^C‐regulated maternal mRNA stabilization is essential for MZT transition.

## Graphical abstract

1. Loss of Nsun5 affects Maternal mRNA stability

2. Nsun5 deficiency influences the translation efficiency of Mad2l2 and Gdf9

3. Nsun5 knockout triggers aberrant events in the exon region of Brd8

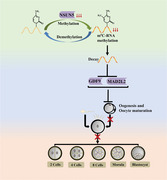

## 1 INTRODUCTION

The maternal‐to‐zygotic transition (MZT) transition of mammals is a critical and essential process involving the fusion of two differentiated gametes (sperm and oocyte), eventually resulting in a totipotent zygote state.^1^, ^2^ Throughout this fundamental developmental time, preimplantation embryos go through dramatic change from a maternal‐dominated state to a zygotic‐driven state. In particular, selective renewal and diverse modifications of maternal mRNAs are essential for MZT transition and gametogenesis.^3^, ^4^ Meanwhile, the processes of mRNA stability, export and maturation have been found to be regulated by RNA modification and related alternative splicing.^5^, ^6^, ^7^ For instance, N^6^‐methyladenosine (m^6^A) promotes the degradation of maternal RNA during the MZT process in zebrafish,^3^ and N^6^‐methyladenosin could also manage the maintenance of maternal RNA in mouse oocytes and timely RNA decay during the MZT.^8^

Recent studies have shown that m^6^A in regulated exons modulates alternative splicing via the interaction of m^6^A reader binding and RNAPII occupancy,^9^ while 3′ untranslated region (3′UTR) m^6^A modification correlates with alternative polyadenylation (APA).^10^, ^11^ In addition, m^6^A readers and writers were proven to be key components in oogenesis and initial embryogenesis. Loss of the N^6^‐methyladenosine reader YTHDC1 causes pre‐mRNA transcript defects, which lead to oocyte dysfunction.^12^ Deletion of the m^6^A writer Kiaa1429 leads to abnormal oogenesis in the germinal vesicle breakdown (GVBD) stage, which is due to changes in exon skipping during alternative splicing events.^13^ These findings indicate that RNA methylation modification and its regulators are important for oocyte maturation as well as the MZT transition. However, the detailed molecular mechanisms of RNA posttranscriptional regulation from oogenesis to embryonic development remain largely unknown.

In addition to N^6^‐methyladenosine, another important and longest‐known RNA methylation modification is 5‐methylcytosine (m^5^C). Earlier research revealed that m^5^C exists in various RNA species. For example, m^5^C in tRNAs usually affects RNA stability related to translational regulation.^14^ Additionally, m^5^C in ribosomal RNA (rRNA) impacts the quality control of ribosome biogenesis.^15^ Importantly, 5‐methylcytosine sites are distributed across all regions of mRNA and are especially enriched in the Sequence coding for aminoacids in protein (CDS) and 3′ untranslated region (3′UTR).^16^ Interestingly, m^5^C sites are also primarily situated in CDS with the highest enrichment peak around the stop codon region.^17^ Functionally, m^5^C on mRNA has been shown to impact multiple physiological events, such as gametogenesis and embryonic development.^18^ Dynamic m^5^C methylation evolved an important role in marking and modulating maternal mRNA, subsequently influencing oocyte maturation and MZT.^19^ It is worth noting that NSUN2 and NSUN6 methylate the vast majority of maternal mRNAs to regulate MZT or other mechanisms.^19^ Previous reports indicated that m^5^C affects dynamic maternal mRNA stability in the transcriptome via Ybx1 protein recognition, and m^5^C‐induced RNA decay of maternal mRNAs coordinates temporal and spatial changes during embryogenesis and embryo development.^4^ Obviously, the intricate regulatory mechanisms between the m^5^C writer and the MZT transition still require further study.

The methyltransferase NSUN5 (a member of the NOL1/Nop2/sun protein family) is an enzyme in the m^5^C writer family that modifies rRNA with 5‐methylcytosine. Oxidative stress‐induced NSUN5 loss was found to promote structural changes in 28S/25S rRNA, resulting in regulatory effects on lifespan.^15^ Regarding m^5^C mRNA modification, recent research has shown that NSUN5 reduction weakens retinoic acid‐inducible gene I (RIG‐I) signaling initiated upon viral infection.^20^ In addition, knockdown of NSUN5 triggers cell cycle arrest through inhibition of CDK4 and CDK6 expression. However, the developmental contribution of the m^5^C mRNA‐NSUN5‐mediated mRNA stability pathway to oogenesis and ovarian aging remains unknown.

In this research, our results revealed that NSUN5 deletion leads to the inhibition of ovarian function and the arrest of embryonic development. BS‐m^5^C‐seq data showed that a low level of m^5^C caused by NSUN5 knockout accelerated ovarian aging in a time‐dependent manner. Furthermore, NSUN5 loss induced decreases in MAD2L2 and GDF9 protein levels. Further study revealed that two m^5^C sites in the exon region of *Mad2l2* and one m^5^C site in the 3′UTR of *Gdf9* were correlated with mRNA stability. Overall, NSUN5 regulates the process of alternative splicing in the CDS region during ovarian aging.

## 2 METHODS

### 2.1 Construction of the *Nsun5* ^KO^ mouse model

Mice were maintained in the Animal Research Center of Nanjing Medical University. Our previous study showed that the CRISPR/Cas9 genome editing system was employed to knockout exon 3 of *Nsun5* to generate *Nsun5* ^KO^ mice.^21^ Genomic DNA extracted from mouse tails was used for genotyping. The sgRNA expression plasmids were annealed and cloned into the BsaI sites of pUC57‐sgRNA (Addgene 51,132). The oligo sequences are as follows:

sgRNA1‐sense: TAGGCCCAGCAGAGCCTTCCAT
sgRNA1‐antisense: AAACATGGAAGGCTCTGCTGGG
sgRNA2‐sense: TAGGCTGAGCTGGCCCGACTCA
sgRNA2‐antisense: AAACTGAGTCGGGCCAGCTCAG.

### 2.2 Ovarian follicle count

Five sections of approximately 5 μm thickness were collected from each ovary of mice. After staining with hematoxylin and eosin (HE) and examination of the ovarian structure and follicle phenotype, oocytes containing every follicle were counted once to avoid recounting. The results are presented as the fold change ± SD (standard deviation).

### 2.3 Enzyme‐linked immunosorbent assay

For the hormone assay, .5‐ml blood samples were collected from mice. Following the enzyme‐linked immunosorbent assay kits manufacturer's instructions, the serum levels of Anti‐Müllerian hormone (AMH), follicle‐stimulating hormone (FSH) and 17ß‐estradiol (E2) were detected. Each sample was detected using three wells, and the results are shown as the fold change ± SD.

### 2.4 Western blot analysis

Lysis buffer (Beyotime Biotechnology, China) was used to lyse ovarian cells or human granular cells (hGCs). Twenty micrograms of extracted protein was loaded onto 10% sodium dodecyl sulfate polyacrylamide gel electrophoresis (SDS‐PAGE) gels, and the separated proteins were electroblotted onto PVDF membranes. The membranes were incubated with primary antibodies (Table S2) at 4°C overnight, followed by incubation with secondary antibodies at room temperature for 2 h. The protein signal from each sample was detected via enhanced chemiluminescence and scanned with a chemiluminescence detection system. Experiments were repeated three times. The results are presented as the fold change ± SD.

### 2.5 Coimmunoprecipitation and immunoblot assay

Immunoprecipitation was performed according to a protocol from Roche Diagnostics. Protein G beads were washed three times with lysis buffer and incubated with 400 μg of tissue lysate together with 4 μg of mouse anti‐NSUN5 or IgG (normal mouse IgG: SC‐3879, Santa Cruz) overnight at 4°C. Finally, the beads were eluted with 5 × SDS sample buffer for immunoblot analysis.

### 2.6 RNA extraction and DNase treatment

Total RNA was extracted from ovarian tissue and the KGN cell line using TRIzol reagent (Invitrogen, 15596018) according to the manufacturer's instructions. Samples with high quality (28S/18S > 2) were selected for further experiments. Turbo DNase (Invitrogen, AM2239) treatment was implemented to remove DNA contamination. A Qubit RNA HS Assay Kit (Thermo Fisher Scientific, Q32855) was used to test the concentration of total RNA.

### 2.7 Quantitative reverse transcription‐polymerase chain reaction

Total RNA was extracted and reverse transcribed into cDNA. Quantitative reverse transcription‐polymerase chain reaction (qPCR) was performed using SYBR premix (Biosharp, BL698A) following the manufacturer's instructions. The primer sequences used are listed in Table S1. Gapdh was as housekeeping gene for this study.

### 2.8 BS‐m^5^C‐seq library construction

mRNA was separated from 12.5 μg of total RNA, and bisulfite was converted using the EZ RNA methylation kit with high‐stringency conditions. Then, RNA libraries were constructed with the NEBNext Ultra II Directional RNA Library Prep Kit (New England Biolabs, United States). An Agilent 2100 was employed to evaluate the library quality, and then the library was sequenced with Illumina NovaSeq.

### 2.9 Identification of m^5^C sites and genes in mRNA

To confirm the presumptive m^5^C sites, clean reads were mapped to the mm10 genome using the meRanGh tool from meRanTK. The m^5^C sites were called using meRanCall from meRanTK, and the R package ChIPseeker was used to annotate the CallResult files obtained by meRanTK. Afterwards, the distribution of m^5^C sites in mRNA was analyzed with the R package Guitar.

### 2.10 Culture of KGN cells

The KGN cell line was cultured in Dulbecco's modified Eagle medium (DMEM) with 10% fetal bovine serum at 37°C with 5% CO_2_.

### 2.11 Oocyte collection and handling

2.11

GV oocytes were isolated from the ovaries of mice, and the cells were released into M2 culture media. Metaphase II (MII) oocytes at 14–16 h were harvested from the oviducts and released into M2 medium at 37°C for 3–5 min.

### 2.12 Analysis of alternative splicing associated with *Nsun5* ^KO^

To obtain alternative splicing information, we used the R package APAlyzer to process the bam file obtained by meRanGh. We used the APAdiff function to calculate the relative expression difference (RED) value.^22^

### 2.13 Ovary and oocyte immunofluorescence

For ovarian immunofluorescence (IF), after pretreatment of paraffin‐embedded mouse ovarian sections, the ovarian sections were incubated with primary antibodies at 4°C overnight and then incubated with secondary antibodies at room temperature for 40 min. The sections were mounted using Hoechst and viewed under a microscope. The images were captured using an Olympus digital camera. Information concerning the primary antibodies used is provided in Table S2. For oocyte IF, GV and MII oocytes were fixed in 4% paraformaldehyde and permeabilized with .5% Triton X‐100. The oocytes were incubated with primary antibodies at 4°C overnight and then incubated with the corresponding secondary antibodies at room temperature (RT) for 2 h. The oocytes were detected under a confocal laser scanning microscope. The antibodies used are listed in Table S2.

### 2.14 Luciferase reporter assay

The KGN cell line was transfected with the pmirGLO luciferase vector (Promega, E1330) fused with wild‐type or mutated Nsun5. A dual‐luciferase reporter assay system was used to test the luciferase activity according to the manufacturer's instructions.

### 2.15 RNA stability assay

Nsun5 shRNA was transfected into human granule cells, and then the cells were seeded into 12‐well plates to achieve 70% confluency after 24 h. Cells were treated with 5 μg/ml actinomycin D and collected at the indicated time points for detection.

### 2.16 Statistical analysis

The values are shown as the mean ± SD. Statistical analysis was performed using GraphPad Prism 8 software using an unpaired t test where appropriate. Probability values <.05 were considered significant.

## 3 RESULTS

### 3.1 *Nsun5^KO^* mouse model establishment and Nsun5 deletion induced decreased ovarian function

**FIGURE 1:**
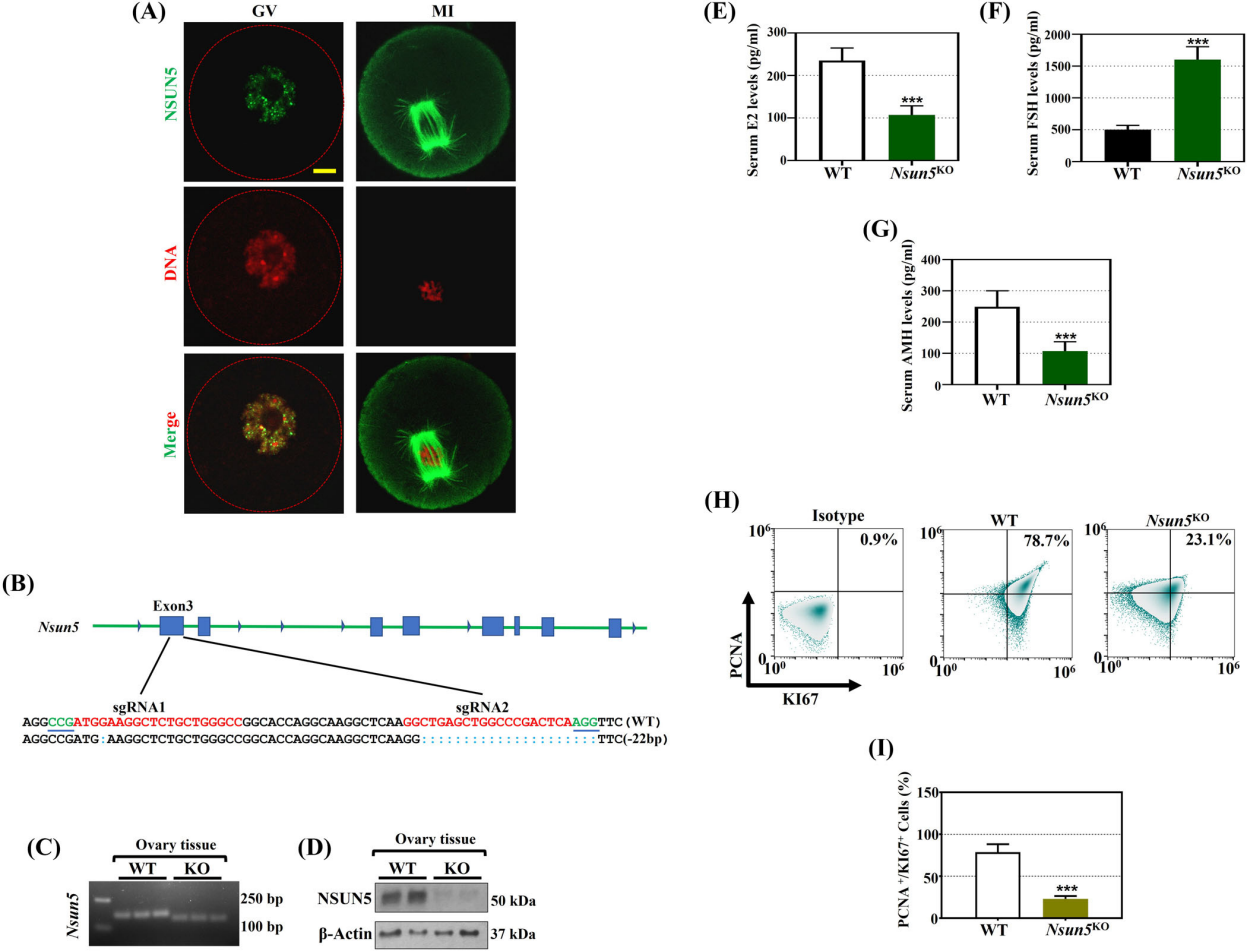
Genotype of NOP2/Sun RNA methyltransferase 5 knockout (*Nsun5* ^KO^) female mice and changes in ovarian function. (A) Fluorescence localization of NSUN5 (green) and nuclear (red) staining in Germinal vesicle (GV), Metaphase II (MII) oocytes. (B) The CRISPR/Cas9 genome editing system for *Nsun5* knockout in mice. (C) *Nsun5* mRNA expression in the *Nsun5* ^KO^ mouse model. (D) NSUN5 protein expression in the *Nsun5* ^KO^ mouse model. (E–G) The serum levels of 17ß‐estradiol (E2), follicle‐stimulating hormone (FSH) and Anti‐Müllerian hormone (AMH) in *Nsun5* ^KO^ and WT mice (***p < .001). (H) FACS analysis was further carried out to assess cell viability in *Nsun5* ^KO^ and WT cells with the proliferation markers PCNA and KI67. (I) The percentage of PCNA‐ and KI67‐positive cells in *Nsun5* ^KO^ mice (***p < .001)

**FIGURE 2:**
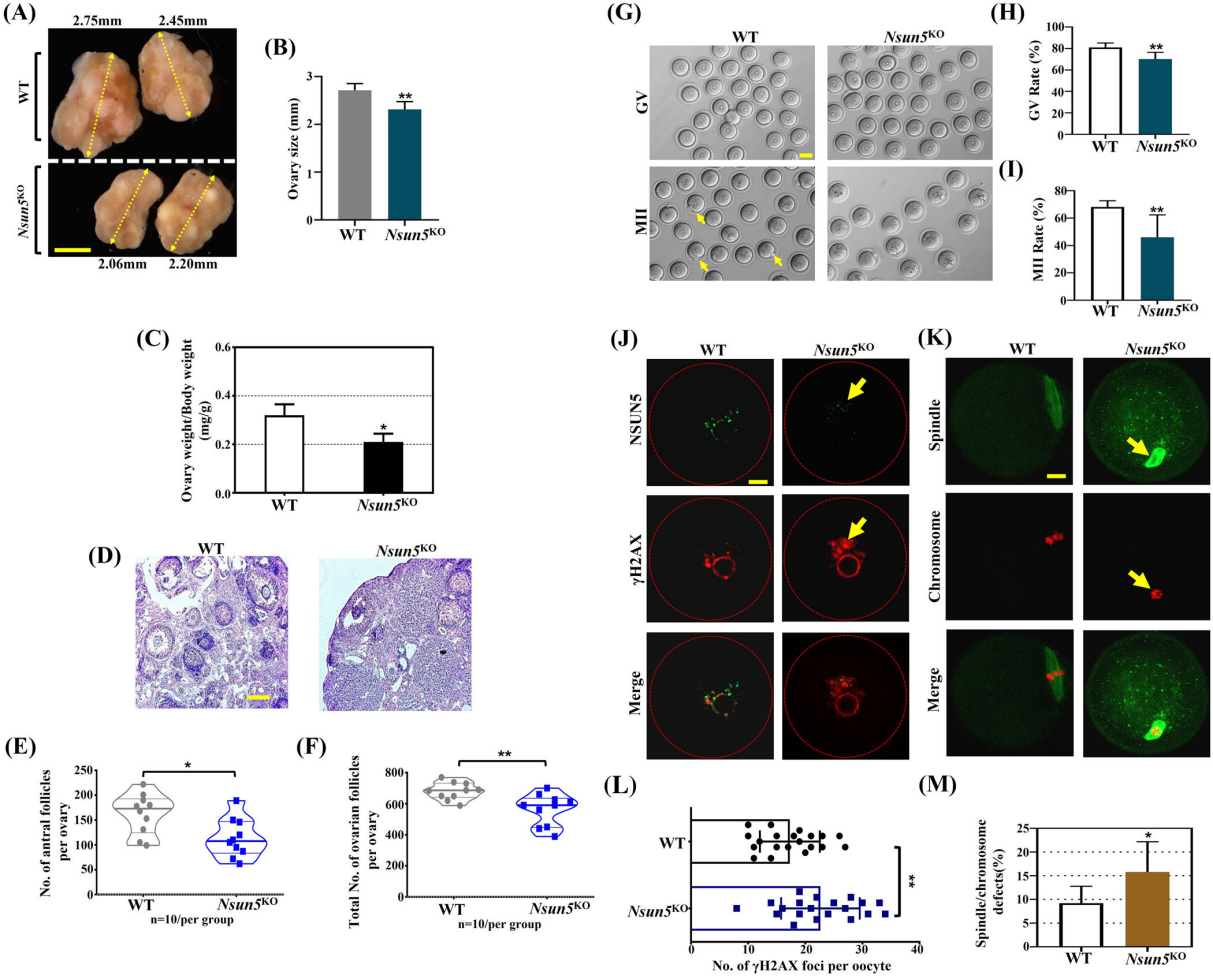
*Nsun5* ^KO^ female mice showed ovarian function decline. (A and B) Macroscopic analysis of ovarian size in *Nsun5* ^KO^ and WT mice. (Scale bars, 500 μm). (C) The ratio of ovary weight to body weight in *Nsun5* ^KO^ and WT mice (*p < .05, *n* = 10). (D) HE staining of *Nsun5* ^KO^ and WT ovaries (scale bars, *n* = 10, 100 μm). (E and F) The average number of antral follicles and total number of follicles in *Nsun5* ^KO^ and WT ovaries (*n* = 10, follicles/per group, *p < .05, **p < .01). (G) Mature germinal vesicle (GV) and MII oocytes in *Nsun5* ^KO^ and WT ovaries (scale bars, 20 μm). (H and I) The maturation rate of GV and MII oocytes in *Nsun5* ^KO^ and WT ovaries (**p < .01, *n* = 10). (J) Left: images of NSUN5 (green) and γ‐H2AX (red) staining in *Nsun5* ^KO^ and WT oocytes. (K) Right: images of spindle (green) and chromosome (red) staining in *Nsun5* ^KO^ and WT oocytes. (L) The number of γ‐H2AX foci per oocyte in *Nsun5* ^KO^ and WT mice (*n* = 20, **p < .01). (M) The percentage of spindle and chromosome defects in *Nsun5* ^KO^ and WT oocytes (*n* = 20, *p < .05)

Loss of Nsun5 usually leads to decreased proliferation and size in different mammalian cells.^23^ Consequently, IF confirmed that NSUN5 was mainly localized in the nucleus of the GV and spindle in the meiosis stage (Figure 1A). To understand the role of Nsun5 in the ovary, we generated an *Nsun5* knockout (*Nsun5* ^KO^) mouse model by the CRISPR/Cas9 genome editing system (Figure 1B), and then we confirmed that the expression of Nsun5 was significantly decreased at the mRNA and protein levels (Figure 1C,D). Statistical analysis revealed that the E2 and AMH levels were both significantly reduced, while the FSH level dramatically increased in *Nsun5* ^KO^ mice compared to WT mice (Figure 1E–G). Moreover, fluorescence activated cell sorting (FACS) showed that the proliferation rate of PCNA‐ and KI67‐positive *Nsun5* ^KO^ cells significantly decreased to 23.1% (Figure 1H,I). Phenotypic analysis presented ovarian organ size differences: *Nsun5* ^KO^ mice (2.06 mm, 2.20 mm) were significantly smaller than WT mice (2.75 mm, 2.45 mm) (Figure 2A,B). In addition, the ratio of ovary weight to body weight in *Nsun5* ^KO^ mice was evidently lower than that in WT mice (Figure 2C). Furthermore, HE staining showed that the number of follicles in *Nsun5* ^KO^ ovaries was substantially decreased compared to that in WT ovaries (Figure 2D). Specifically, the average number of antral follicles and total follicles in *Nsun5* ^KO^ ovaries was markedly decreased compared to that in WT ovaries (Figure 2E,F).

Importantly, we noticed that the maturation rates of GVBD and MII oocytes were both significantly lower in *Nsun5* ^KO^ oocytes than in WT oocytes (Figure 2G–I, 81% maturation rate of GVBD in WT vs. 70% in *Nsun5* ^KO^ oocytes, 68% maturation rate of MII oocytes in WT vs. 46% in *Nsun5* ^KO^ oocytes). As expected, the *NSUN5* signal was reduced in *Nsun5* ^KO^ oocytes. To illustrate the role of *NSUN5* in early oocyte development, IF was performed, and the images revealed an increase in the number of DNA damage marker γ‐H2AX foci per oocyte in *Nsun5* ^KO^ mice compared to that in WT mice (Figure 2J,L). We found a higher percentage of spindle and chromosome defects in *Nsun5* ^KO^ oocytes than in WT oocytes (Figure 2K,M, 15% vs. 9%).

### 3.2 *Nsun5* ^KO^ in female mice induced embryonic development retardation

**FIGURE 3:**
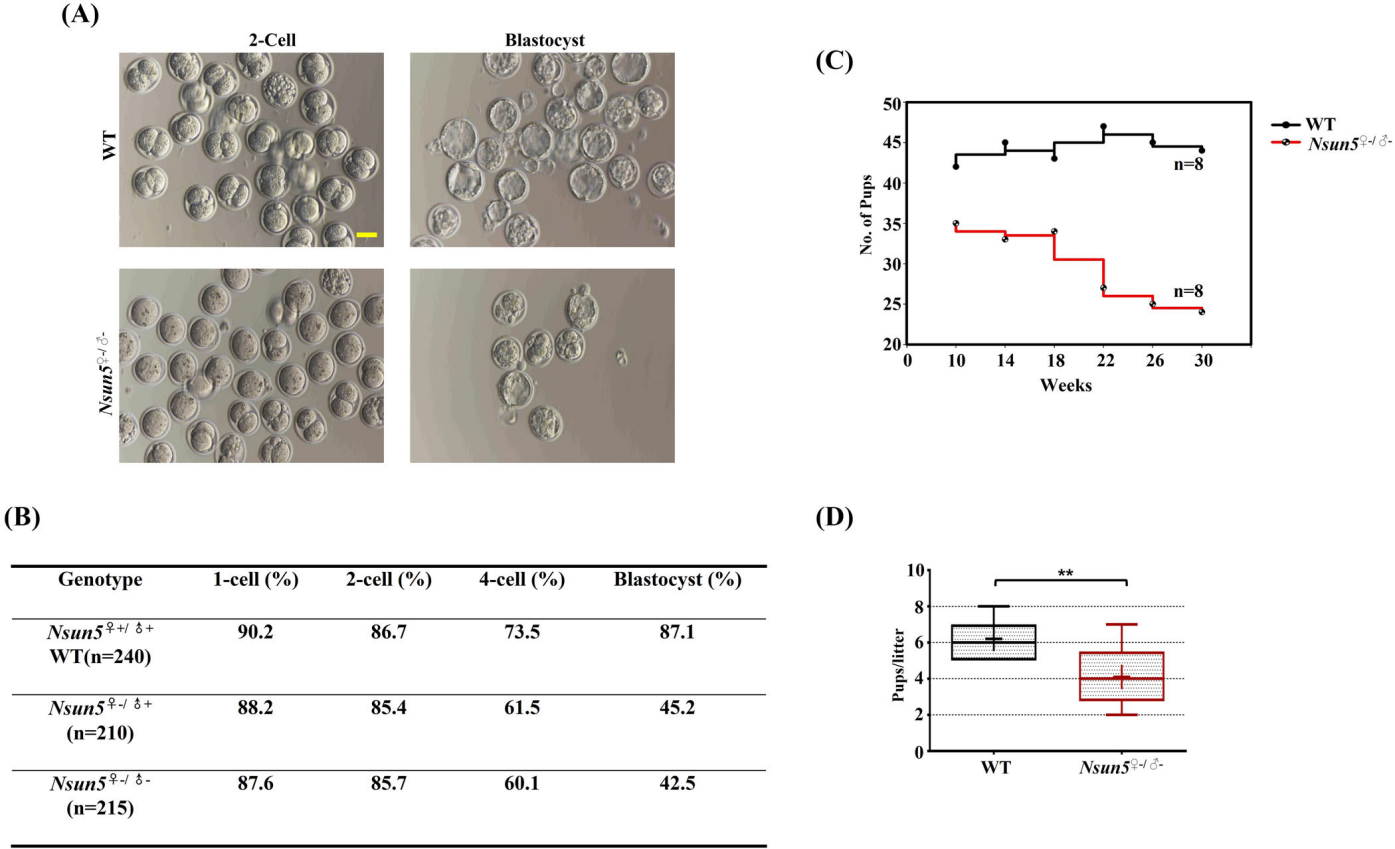
*Nsun5* ^KO^ influenced the developmental potential in embryos. (A) Mature 2‐cell embryos and blastocysts in *Nsun5* ^KO^ and WT mice (scale bars, 100 μm). (B) The percentage of embryonic development in the three Nsun5 genotype groups (*Nsun5* ^♀+/♂+^, *Nsun5* ^♀‐/♂+^, and *Nsun5* ^♀‐/♂−^) (C). The mouse litter number in *Nsun5* ^♀‐/♂‐^ and WT mice from 0 to 30 weeks (*n* = 8). (D) The number of pups per litter in *Nsun5* ^♀‐/♂‐^ and WT mice (**p < .01)

To investigate the contribution of Nsun5 to the MZT transition, our results indicated that the number of embryos from *Nsun5* ^KO^ mice was markedly lower than that from WT mice at the blastocyst stages (Figure 3A). In addition, we monitored the percentage of embryo development in three Nsun5 genotype groups (*Nsun5* ^♀+/♂+^, *Nsun5* ^♀‐/♂+^ and *Nsun5* ^♀‐/♂−^) at four stages (1‐cell, 2‐cell, 4‐cell and blastocyst). The results indicated that the percentage of embryogenesis displayed a distinct pattern in which the proportion of normal embryos decreased from 1‐cell embryos to blastocysts in the *Nsun5* ^♀‐/♂+^ group (from 88.2% to 45.2%) and in the *Nsun5* ^♀‐/♂−^ group (from 87.6% to 42.5%) (Figure 3B and Figure S1). Similarly, *Nsun5* ^♀‐/♂‐^ mice presented poor fertility, since the number of pups per mouse gradually decreased with age, especially from 18 to 26 weeks, while the opposite tendency was observed in WT mice (Figure 3C). The number of pups per litter in *Nsun5* ^♀‐/♂‐^ mice was decreased compared to that in WT mice (Figure 3D). These results implied that maternal Nsun5 RNA is crucial for embryo development and fertility maintenance.

Overall, these results suggest that Nsun5 is involved in embryogenesis during different developmental stages.

### 3.3 BS‐m^5^C‐seq discovered *Nsun5* target genes

**FIGURE 4:**
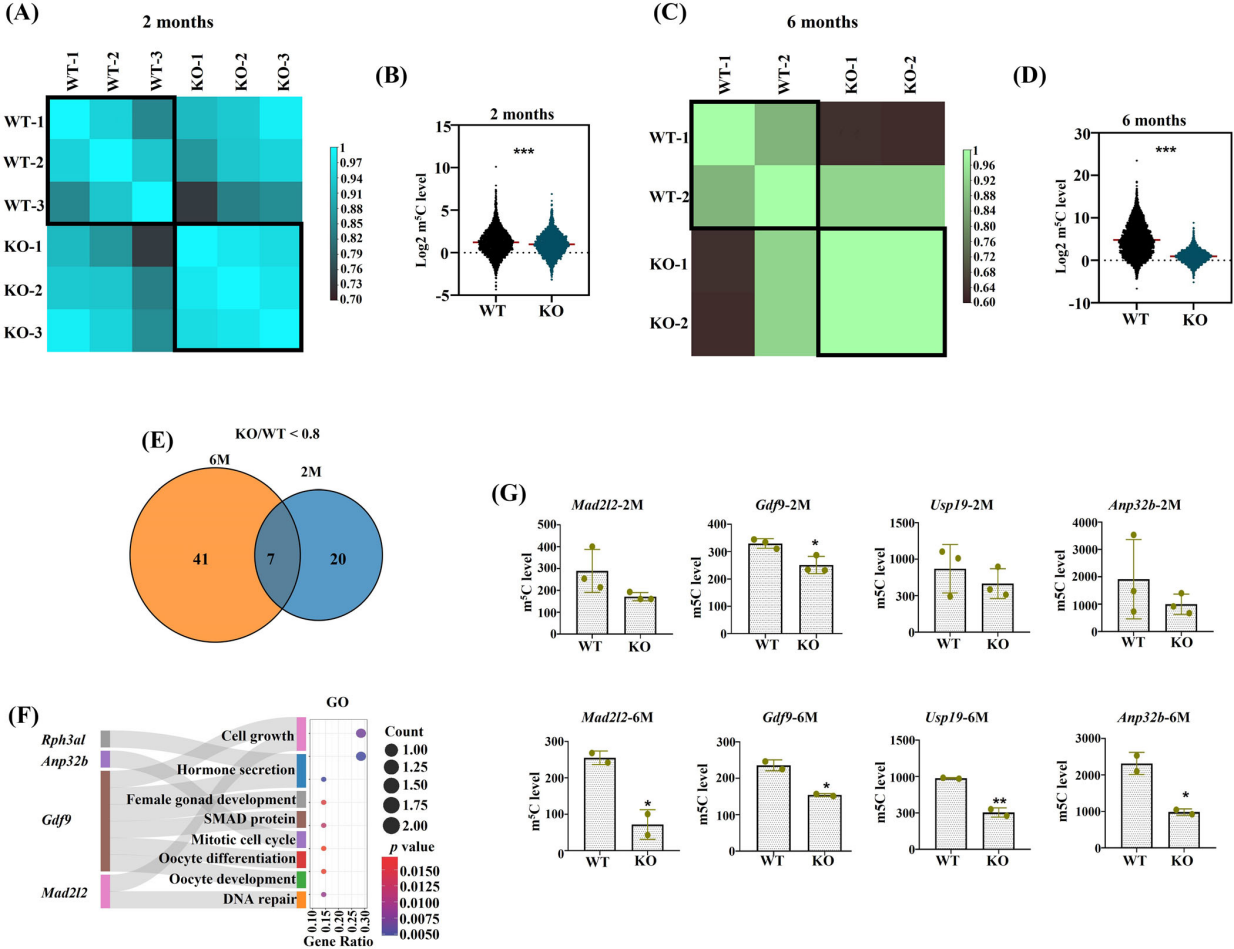
Bioinformatic analysis demonstrated the change in m^5^C modification during ovarian aging after *Nsun5* ^KO^. (A) Heatmap of the Pearson correlations of the m^5^C level of matched *Nsun5* ^KO^ and WT ovaries at 2 months. (B) Violin plot showing that the overall m^5^C level was significantly decreased in the *Nsun5* ^KO^ group compared to the WT group at 2 months (***p < .001). (C) Heatmap of the Pearson correlations of the m^5^C level of matched *Nsun5* ^KO^ and WT ovaries at 6 months. (D) Violin plot showing that the overall m^5^C level was significantly decreased in the *Nsun5* ^KO^ group compared to the WT group at 6 months (***p < .001). (E) Venn diagram showing the overlap of m^5^C genes with the ratio (KO/WT < .8) between 2 and 6 months. (F) GO enrichment analysis of the seven overlapping m^5^C genes. (G) The m^5^C levels of *Usp19*, *Anp32b*, *Mad2l2* and *Gdf9* in the *Nsun5* ^KO^ and WT groups at two time points (*p < .05, **p < .01)

To investigate the dynamic pattern of m^5^C sites and genes in the ovaries between *Nsun5* ^KO^ and WT mice, mouse ovarian samples were subjected to BS‐m^5^C‐seq at the 2‐month and 6‐month stages. As predicted, the overall m^5^C levels in *Nsun5* ^KO^ and WT ovarian samples showed high similarity with intragroup (WT1, WT2 group… intercomparison, or KO1, KO2 group… intercomparison) but tended to be more diverse with intergroup (WT and KO group intercomparison) at two and 6 months (Figure 4A and 4C). We noted that the overall m^5^C level significantly decreased in the *Nsun5* ^KO^ group compared with the WT group at 2 months, and the difference was greater at 6 months (Figure 4B and 4D). Although the number of m^5^C genes exhibiting a single m^5^C site in *Nsun5* ^KO^ mice was slightly lower than that in WT mice at two and 6 months, there were almost no differences among the remaining four groups with different sites (sites = 2–5) (Figure S2A and 2C). Then, we selected differentially modified m^5^C genes to perform gene ontology (GO) analysis and found eight important m^5^C regulatory genes (2 months) and eleven genes (6 months) that were enriched in female reproduction pathways (Figure S2B and 2D). According to the standard of the m^5^C value in the *Nsun5* ^KO^ group divided by the m^5^C value in the WT group, the specific value was less than .8, we investigated the molecular mechanism and discovered seven overlapping m^5^C genes between the two groups (Figure 4E). Next, we performed GO enrichment analysis of the seven overlapping m^5^C genes and confirmed that *Mad2l2*, *Gdf9*, *Rrh3al* and *Anp32b* were highly enriched in the top eight pathways (Figure 4F). Furthermore, we calculated the m^5^C levels of *Usp19*, *Anp32b*, *Mad2l2* and *Gdf9* in ovaries from *Nsun5* ^KO^ and WT mice at two stages. We discovered that the m^5^C levels of the four genes were all lowered in *Nsun5* ^KO^ at 2 months, and the difference was greater at 6 months (Figure 4G).

### 3.4 Nsun5 affected oocyte function by inhibiting m^5^C modification of *Mad2l2*

**FIGURE 5:**
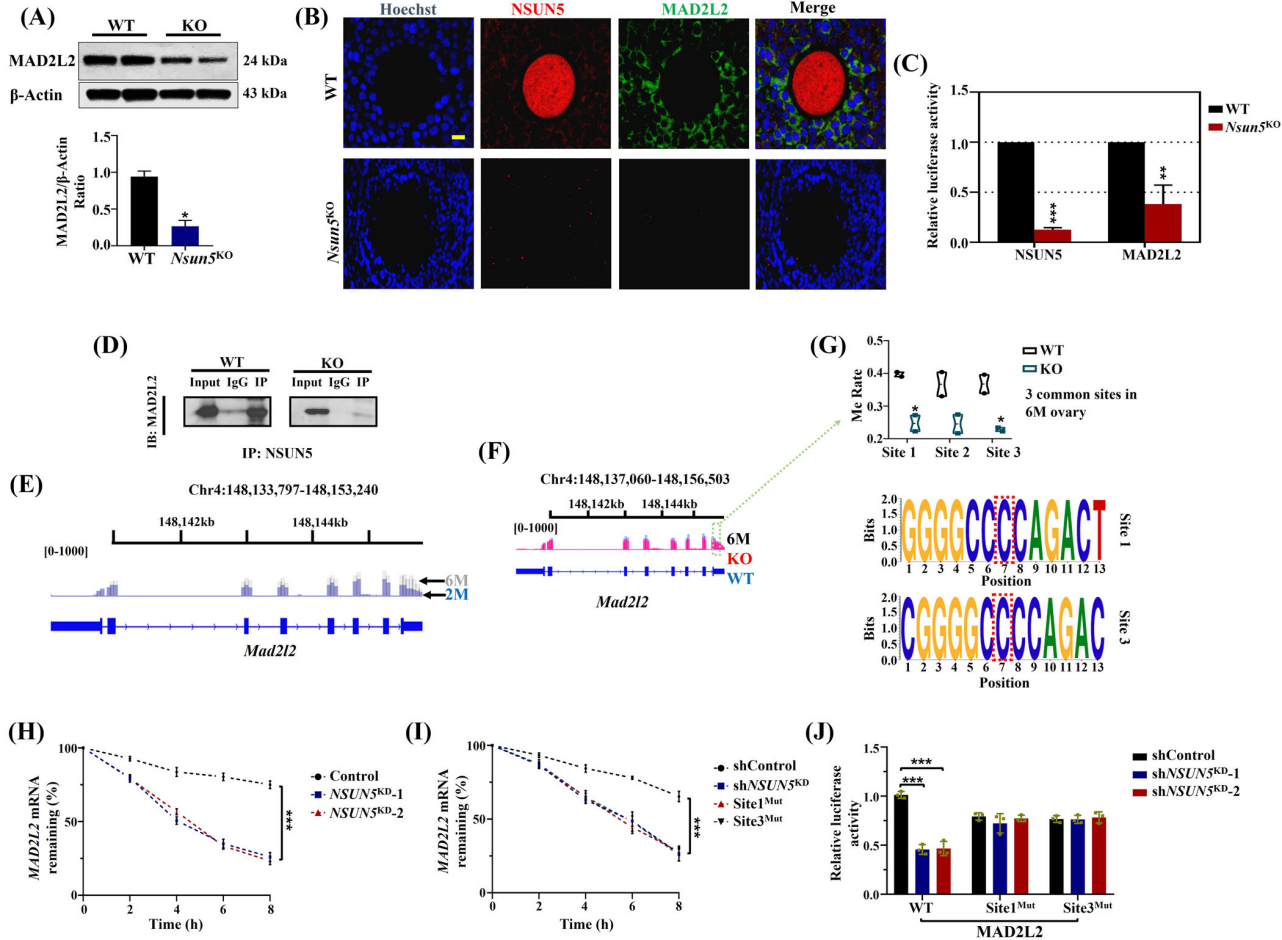
Nsun5 affected oocyte function by inhibiting the m^5^C modification of *Mad2l2*. (B and C) Relative luciferase activity of Hoechst (blue), NSUN5 (red) and MAD2L2 (green) in the *Nsun5* ^KO^ and WT groups (*p < .05, **p < .01). (A) Protein expression of MAD2L2 in the *Nsun5* ^KO^ and WT groups (*p < .05). (D) Co‐immunoprecipitation (Co‐IP) showing the interaction between NSUN5 and MAD2L2. (E) Representative m^5^C sites of *Mad2l2* on chromosome 4 in the WT group. (F) Representative m^5^C sites of *Mad2l2* on chromosome 4 at 6 months in the Nsun5^KO^ and WT groups. (G) Differential methylation rates of two common m^5^C sites at 6 months (*p < .05). (H) *MAD2L2* mRNA stability of *NSUN5* ^KD^ and control KGN cells (***p < .001). (I) mRNA stability of *MAD2L2* in the control, *NSUN5* ^KD^, site 1^Mut^ and site 3^Mut^ groups (***p < .001). (J) Luciferase expression of MAD2L2‐WT, MAD2L2‐site 1^Mut^ and MAD2L2‐site 3^Mut^ in the control and *NSUN5* ^KD^ cell lines (***p < .001)

To validate the sequencing results, Western blotting and IF were carried out to confirm that the protein expression of MAD2L2 protein was significantly reduced in the ovaries of *Nsun5* ^KO^ mice (Figure 5A–C). To verify the interaction between NSUN5 and MAD2L2, co‐immunoprecipitation (Co‐IP) was performed, and compared with the visible bands for NSUN5 in WT mice, the Co‐IP band was nearly absent in *Nsun5* ^KO^ mice (Figure 5D). These results implied that there is a direct relationship between NSUN5 and MAD2L2. Interestingly, *Mad2l2* showed m^5^C site patterns between the two stages in WT mice: more m^5^C sites at 2 months overlapped with 6 months (Figure 5E). Hence, we further analyzed the m^5^C sites of *Mad2l2* in the exon region across chromosome 4 in *Nsun5* ^KO^ mice (Figure 5F) and found that the methylation rate of two common m^5^C sites was significantly decreased in *Nsun5* ^KO^ ovaries at the 6‐month stage compared with WT ovaries (Figure 5G). Next, site 1 and site 3, which showed significant differences, were chosen for further *NSUN5* knockdown and site mutation experiments in the KGN cell line. We discovered that NSUN5 knockdown clearly decreased *MAD2L2* mRNA stability (Figure 5H). Mutation of site 1 or site 3 of *MAD2L2* impacted *MAD2L2* mRNA stability (Figure 5I). In addition, luciferase expression of MAD2L2‐WT strongly decreased in *NSUN5* ^KD^ cells, whereas mutation of site 1 or site 3 in MAD2L2 had no such effect (Figure 5J).

### 3.5 Nsun5 affected ovarian function by inhibiting m^5^C modification of *Gdf9*

**FIGURE 6:**
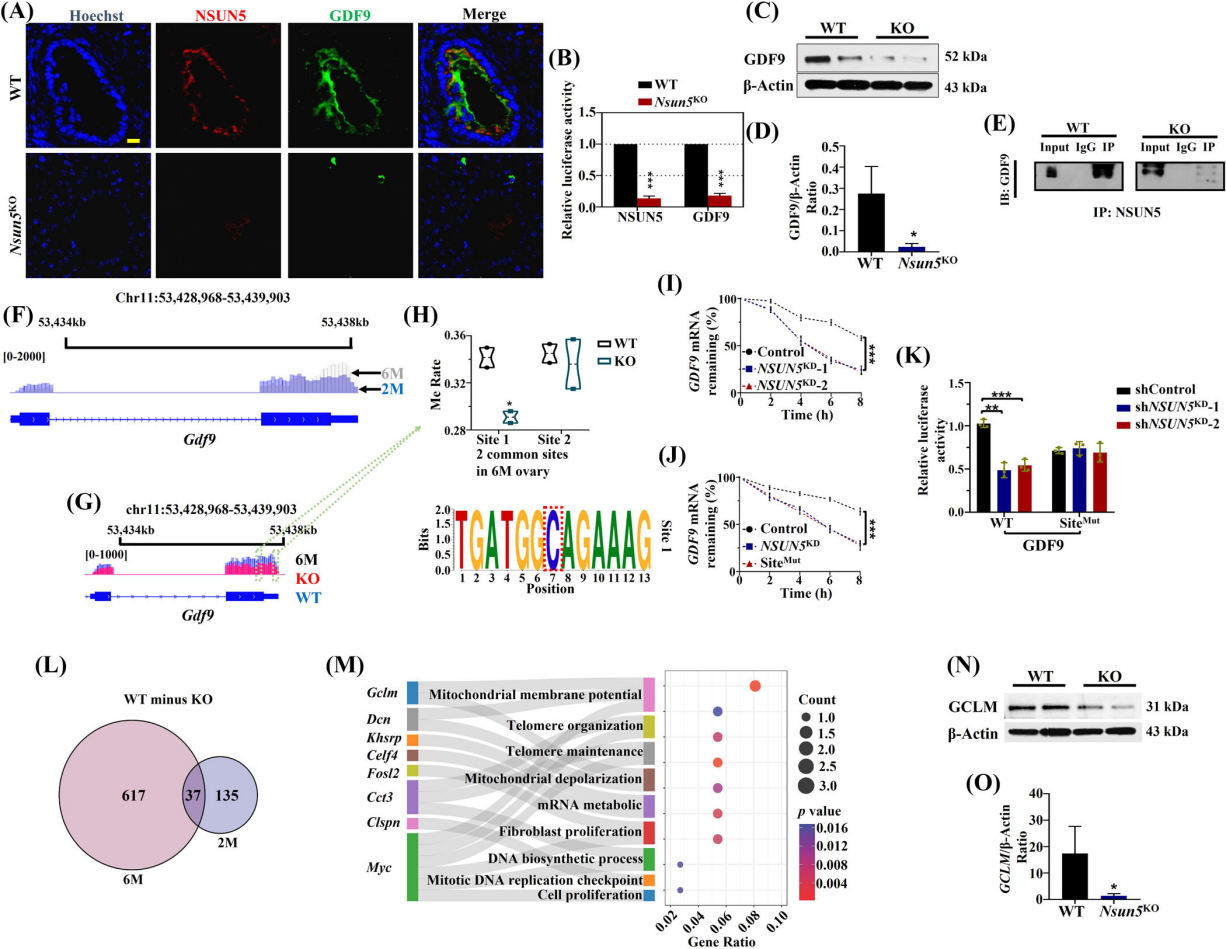
Nsun5 affected ovarian function by inhibiting m^5^C modification of *Gdf9*. (A and B) The relative luciferase activity of Hoechst (blue), NSUN5 (red) and GDF9 (green) in the *Nsun5* ^KO^ and WT groups (***p < .001). (C and D) The GDF9/β‐actin ratio in the *Nsun5* ^KO^ and WT groups (*p < .05). (E) Co‐immunoprecipitation (Co‐IP) showing an intense interaction between NSUN5 and GDF9. (F) Representative m^5^C sites of *Gdf9* on chromosome 11 in the WT group ovaries at two stages. (G) Representative m^5^C sites of *Gdf9* on chromosome 11 in the *Nsun5* ^KO^ and WT groups at 6 months. (H) The methylation rate of differential expression in one common m^5^C site of *Gdf9* at 6 months (*p < .05). (I) *GDF9* mRNA stability of *NSUN5* ^KD^ and control KGN cells (***p < .001). (J) mRNA stability of *GDF9* in the control, *NSUN5* ^KD^ and site 1^Mut^ groups (***p < .001). (K) Luciferase expression of GDF9‐WT and GDF9‐site 1^Mut^ in the control and *NSUN5* ^KD^ cell lines (***p < .001). (L) Venn diagram showing the overlap of m^5^C genes only in WT mice between two and 6 months. (M) GO analysis of 37 overlapping m^5^C genes. (N and O) The protein expression of glutamate cysteine ligase (GCLM) in *Nsun5* ^KO^ and WT ovaries

GDF9 is an oocyte‐specific member of the transforming growth factor#x02010;β (TGF‐β) superfamily and controls granulosa cell growth and differentiation.^24^ Hence, we carried out IF to examine the NSUN5 and GDF9 signals in *Nsun5* ^KO^ and WT oocytes (Figure 6A). Similar to MAD2L2, the luciferase activity of GDF9 and NSUN5 dramatically decreased in *Nsun5* ^KO^ oocytes (Figure 6B). Additionally, the protein expression of GDF9 was significantly reduced in *Nsun5* ^KO^ mouse ovaries (Figure 6C,D). In addition, we performed Co‐IP experiments for NSUN5 and GDF9, and the results showed an intense association between NSUN5 and GDF9 (Figure 6E). Importantly, more m^5^C sites at 2 months overlapped with 2 months in WT mouse ovaries across *Gdf9* on chromosome 11 in the 3′UTR (Figure 6F). Furthermore, the m^5^C site value of *Gdf9* in *Nsun5* ^KO^ mice was much lower than that in WT mice (Figure 6G). Further study showed that the methylation rate of one common m^5^C site was substantially decreased in *Nsun5* ^KO^ ovaries compared to WT ovaries at the 6‐month stage (Figure 6H). Moreover, *GDF9* mRNA stability quickly declined not only after *NSUN5* knockdown (Figure 6I) but also after site 1 mutation of *GDF9* (Figure 6J). Additionally, luciferase expression of GDF9‐WT was strongly reduced in Nsun5^KD^ cells, but site 1 mutation of GDF9 had no effects (Figure 6K). Next, the unique m^5^C genes in *Nsun5* ^KO^ mouse ovaries were identified (The genes have m^5^C value in the WT group were selected, m^5^C value of these genes in the *Nsun5* ^KO^ group at “0”). There were 37 m^5^C genes that overlapped in the 6‐month and 2‐month groups (Figure 6L). The GO analysis of 37 overlapping m^5^C genes revealed that glutamate cysteine ligase (*Gclm*), a marker related to oxidative stress, was enriched in mitochondrial membrane and mitochondrial depolarization (Figure 6M). The protein expression of GCLM was strongly reduced in *Nsun5* ^KO^ ovaries (Figure 6N,O).

In summary, Nsun5‐mediated m^5^C modification could affect ovarian development and aging, while MAD2L2 and GDF9 cooperated with Nsun5 to impact the functions of the mouse ovary.

### 3.6 *Nsun5* ^KO^ induced exon skipping of the *Brd8* transcript to regulate ovarian function

**FIGURE 7:**
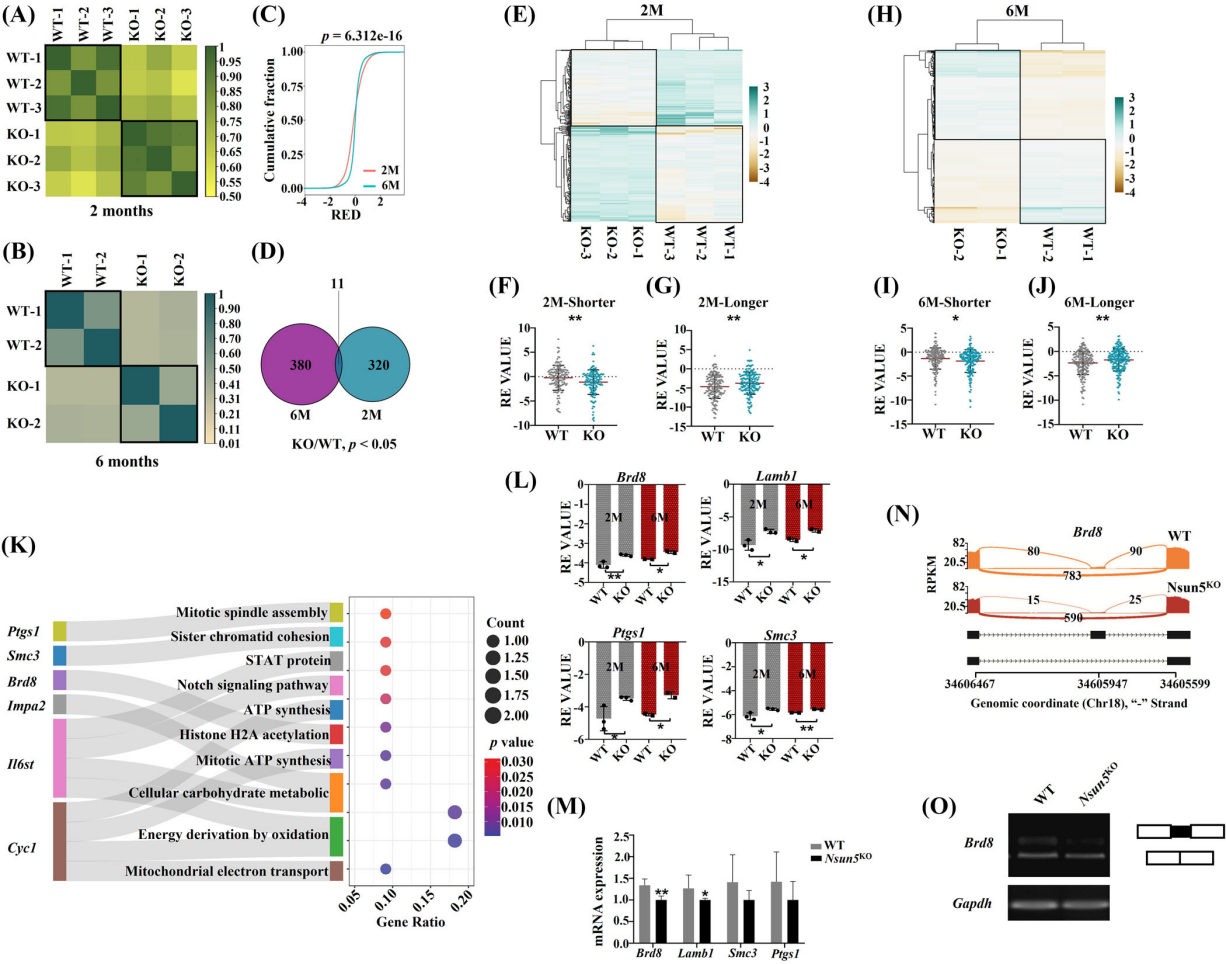
Aberrant alternative splicing (AS) in *Nsun5* ^KO^ ovaries. (A and B) Heatmap of the Pearson correlations of gene Relative expression difference (RED) values from Nsun5^KO^ and WT ovaries at 2 and 6 months. (C) Cumulative fraction plot showing the tendency of the gene RED values at two and 6 months. (D) Venn diagram showing the overlap of AS genes with the ratio (KO/WT < .8, *p* < .05). (E) Heatmap of the pearson correlations of 320 AS genes at 2 months. (F and G) Violin plot showing shorter and longer gene Relative expression (RE) values at 2 months (**p < .01). (H) Heatmap of the Pearson correlations of 380 AS genes at 6 months. (I and J) Violin plot showing shorter and longer gene RE values at 6 months. (K) GO analysis of eleven overlapping AS genes. (L) Scatterplots showing the RE values for *Brd8*, *Lamb1*, *Ptgs1* and *Smc3* at two stages (*p < .05, **p < .01). (M) The mRNA expression of *Brd8*, *Lamb1*, *Ptgs1* and *Smc3* in *Nsun5* ^KO^ and WT mouse ovaries at 6 months (*p < .05, **p < .01). (N) A representative Sashimi plot showing the exon skipping event for *Brd8* in *Nsun5* ^KO^ and WT ovaries. (O) RT‐quantitative reverse transcription‐polymerase chain reaction (qPCR) confirming the exon skipping event for *Brd8* exon 16 in *Nsun5* ^KO^ ovaries

To investigate differential APA of m^5^C genes in *Nsun5* ^KO^ and WT mouse ovaries, we generated the average RE or RED values of genes from mouse ovarian samples at the two‐ and 6‐month stages.^22^ Interestingly, the RE values presented high intragroup similarity in the *Nsun5* ^KO^ or WT mouse ovary group at the two stages (Figure 7A,B). However, the intergroup correlation coefficient between *Nsun5* ^KO^ and WT mouse ovaries was more significant from 2 to 6 months (Figure 7A,B). Next, we noticed that the cumulative fraction of the RED value revealed significant differences between 2 and 6 months (Figure 7C). Afterwards, we selected the RED value with the ratio (KO/WT, *p* < .05) and found that there were 11 overlapping genes between 2 and 6 months (Figure 7D). Next, we clustered the 320 and 380 differentiated genes into heatmaps. Similarities within each group and differences between different groups were observed (Figure 7E and 7H). Furthermore, we compared shorter and longer RE values between *Nsun5* ^KO^ and WT mouse ovaries at two stages. The shorter and longer RE values at two and 6 months were significantly different between the Nsun5^KO^ and WT groups (Figure 7F,G,I,J). We further used 11 previously overlapping genes to perform GO analysis and found that six crucial genes regulated by *Nsun5* were enriched in 10 important reproduction‐related pathways (Figure 7K). Of the previous six enriched regulatory genes in alternative splicing events, we found that the RE values of ovarian function genes (*Brd8*, *Lamb1*, *Ptgs1* and *Smc3*) between the *Nsun5* ^KO^ and WT groups at the two stages were all significantly different (Figure 7L). Additionally, the mRNA expression levels of *Brd8* and *Lamb1* were considerably decreased between the *Nsun5* ^KO^ and WT groups (Figure 7M). Notably, *Brd8*, an important factor related to histone H2A acetylation, showed aberrant exon skipping events at exon 16 in *Nsun5* ^KO^ mouse ovaries compared with WT mouse ovaries (Figure 7N). RT‐PCR confirmed the decreased expression of *Brd8* exon 16 in *Nsun5* ^KO^ mouse ovaries (Figure 7O). In particular, we selected 391 previously differentiated RE genes at 6 months (Figure 7D) to carry out GO analysis and found that 56 genes were enriched in ten reproductive pathways (Figure S3A). In particular, *Smc3* was involved in the spindle assembly pathway, while *Ythdf2* and *Cnot6 L* were associated with mRNA destabilization, and *Brd8* was related to chromatin and histone modification (Figure S3A). Simultaneously, 56 genes were used to generate a clustering correlation heatmap with statistical analysis and correlation coefficients (Figure S3B). Finally, the RE value of *Brd8* showed significant differences among *Cdkn1b*, *Ythdf2*, *Cnot6l* and *Smc3* (Figure S3C).

Altogether, the results described above suggest that *Nsun5* ^KO^ regulates pre‐mRNA alternative splicing events via m^5^C modification during ovarian aging. *Brd8* may act as a target gene for *Nsun5* that participates in controlling m^5^C modification of chromatin or histones during the growth and aging of oocytes.

## 4 DISCUSSION

The Rev7 mutant induces Mad2 deficiency, mediates mitotic arrest and accumulates DNA damage, which increases the frequency of spindle aberrations and chromosome lags.^25^ In our study, Nsun5 knockout increased the rate of spindle/chromosome defects (Figure 2). Our results suggested a regulatory mechanism by which m^5^C modification deletion decreased the protein level of MAD2L2 and accelerated the loss of *MAD2L2* mRNA stability, which caused a deficiency in ovarian function (Figure 5). Our results showed a direct relationship between m^5^C methylation modification and oocyte maturation. Meanwhile, two functional m^5^C modification sites in *Mad2l2* were discovered (Figure 5). In addition, MAD2L2 deficiency affects Dnmt3b expression and induces cell cycle arrest in primordial germ cells, which is highly relevant to fertility in females.^26^ Our findings indicated that the blastocyst formation rate was decreased by Nsun5 deletion (Figure 3). Therefore, Nsun5 acts as an initiator to regulate the process of embryogenesis and embryo development through the influence of maternal mRNA.

GDF9/BMP15 belongs to the TGF‐β superfamily and are involved in fertilization, female reproductive function and normal ovulation.^27^ GDF9 activity is related to oogenesis and oocyte maturation. Transcription factors of female gametes, such as NOBOX, have been revealed to transactivate the GDF9 promoter, which is vital for granulosa cell and germ cell development.^28^ In our study, the m^5^C‐methyltransferase *Nsun5* ^KO^ inhibited ovarian function by decreasing the number of follicles and reducing the level of hormones (Figure 2). Consequently, the regulation of m^5^C‐modified *Gdf9* by Nsun5 was discovered for the first time. Subsequently, a functional m^5^C modification site in *Gdf9* was identified (Figure 6). One interesting viewpoint on the m^5^C modification was that Nsun5 could affect mRNA stability, contributing to aging‐induced declines in ovarian function. Interestingly, downregulation of the antioxidant gene GCLM leads to an accelerated age‐dependent decrease in the number of primordial follicles through inhibition of glutathione generation.^29^ In our study, *Nsun5* ^KO^ decreased the protein level of GCLM in the mouse ovary, which suggests that deletion of Nsun5 accelerated oxidative DNA damage in oocytes by elevating the level of oxidative damage. Our results support adding FSH to the culture system of granulosa cells and neuroblastoma cells to elevate the protein expression of GCLM.^30^

Pre‐mRNA alternative splicing can result in the coordination of transcriptome and proteome diversity that have distinct biogenesis‐related functions.^31^ Follicle formation and maturation are associated with the regulation of alternative splicing by posttranscriptional modifications of mRNA.^12^ Our results provide new mechanistic insights into *Nsun5* ^KO^‐triggered abnormal exon skipping events of *Brd8* in the ovary, which may contribute to changes in m^5^C modification (Figure 7). A previous study supported our finding that SRSF3 depletion changes AS in Brd8 caused by increased skipping of exons during mouse oocyte meiosis.^32^ In addition, our results indicated that knockdown of Nsun5 promoted an unusual AS process in *Lamb1*, causing inhibition of mRNA expression. In summary, these results support previous findings that abnormal RNA methylation levels cause oocyte development retardation due to changes in alternative splicing of oogenesis genes.^3^, ^4^

**FIGURE 8:**
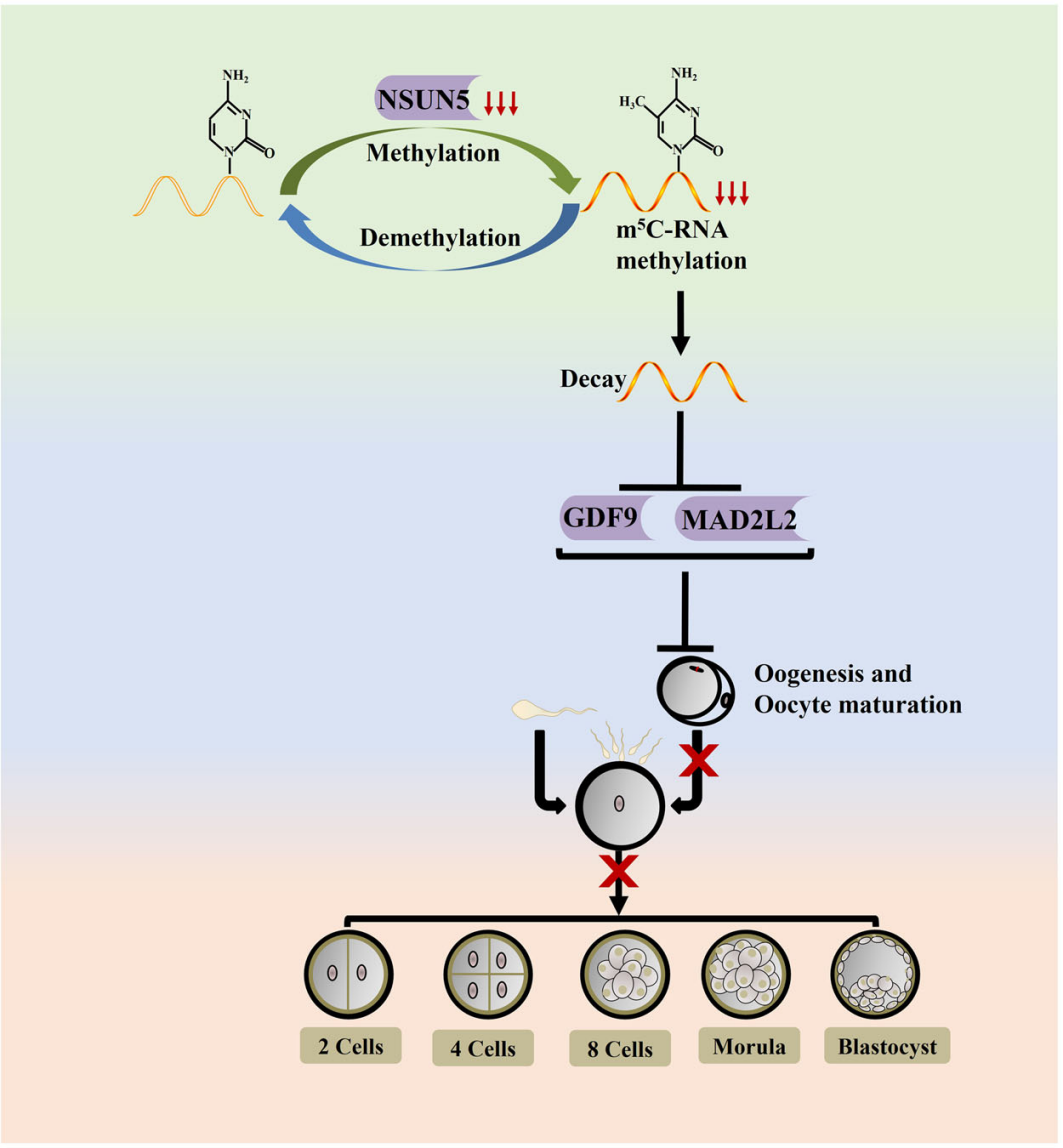
A schematic diagram of the central role of Nsun5 regulation in oocyte aging and embryogenesis

In conclusion, a new mechanistic linkage was found between 5‐methylcytosine and female fertility. Our results show that m^5^C modification of the maternal mRNA of *Mad2l2* and *Gdf9* is specifically regulated by the Nsun5 (Figure 8). In addition, our studies revealed that Nsun5 changed the pattern of alternative splicing in the CDS region during oogenesis and ovarian aging. Overall, we provide convincing evidence that the regulation of mRNA decay and stability by m^5^C modification is essential at multiple stages during the MZT transition.

## CONFLICT OF INTEREST

The authors declare no conflict of interest.

## Supporting information

Supporting Info
