# Supplementary material for: RNA‐methyltransferase Nsun5 controls the maternal‐to‐zygotic transition by regulating maternal mRNA stability
Source: Clin Transl Med. 2022 Dec 10;12(12):e1137. doi: 10.1002/ctm2.1137 (PMC9736783; doi:10.1002/ctm2.1137)
Supplement: Supplementary file 1 — Supporting Info [file CTM2-12-e1137-s001.docx]

**RNA-methyltransferase Nsun5 controls the Maternal-to-Zygotic Transition by regulating Maternal mRNA stability**

**Chenyue Ding ^†^, Jiafeng Lu ^†^, Jincheng Li, Xiujuan Hu, Zhenxing Liu, Han Su, Hong Li *, Boxian Huang** *****

State Key Laboratory of Reproductive Medicine, Suzhou Affiliated Hospital of Nanjing Medical University, Suzhou Municipal Hospital, Gusu School, Nanjing Medical University, Suzhou, 215002, China.

*****Correspondence: [huangboxiannj@163.com](mailto:huangboxiannj@163.com), [hongliszivf@163.com](mailto:hongliszivf@163.com)

**^†^**These authors contributed equally to this work


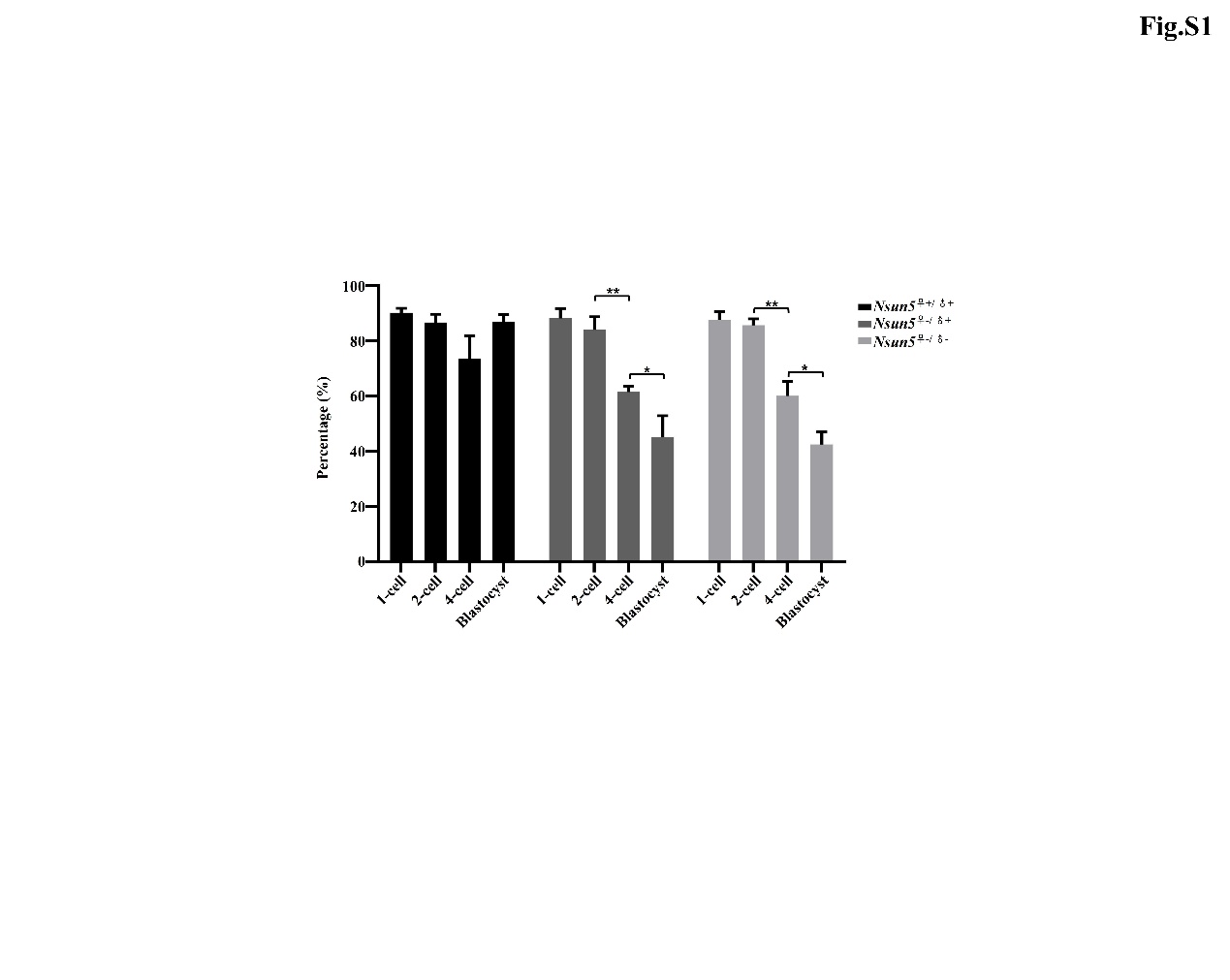


**Figure S1. The percentage of embryogenesis in the *Nsun5*^KO^ homozygosis and heterozygosis group**


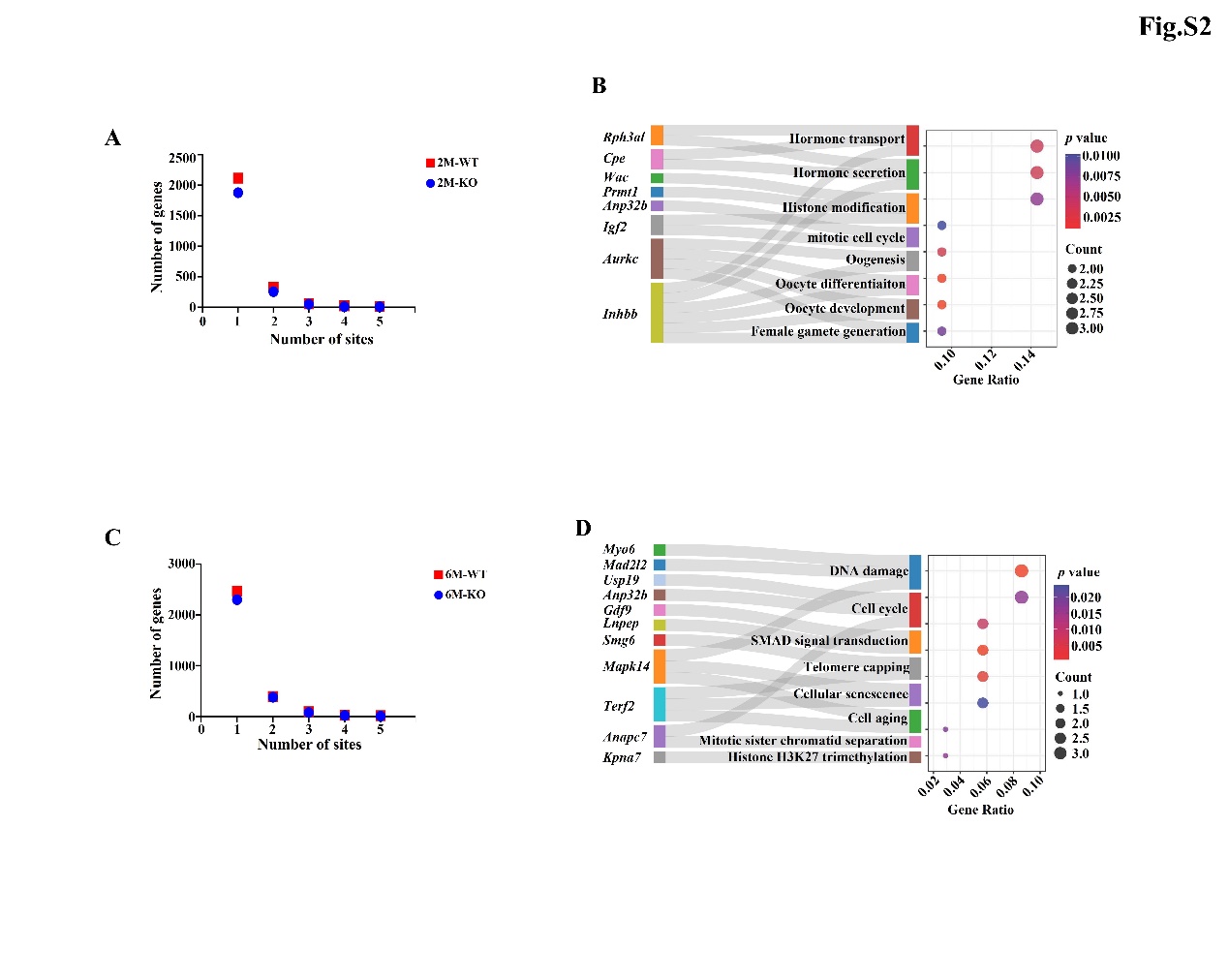


**Figure S2. Dynamic pattern of m^5^C sites and genes between ovaries from *Nsun5*^KO^ and WT mice.**

**A**. The m^5^C genes with different site numbers (sites = 1-5) in *Nsun5*^KO^ and WT mice at two months.

**B**. GO analysis of the top 8 pathways with relevant genes at two months.

**C**. The m^5^C genes with different site numbers (sites = 1-5) in *Nsun5*^KO^ and WT mice at six months.

**D**. GO analysis of the top 8 pathways with relevant genes at six months.


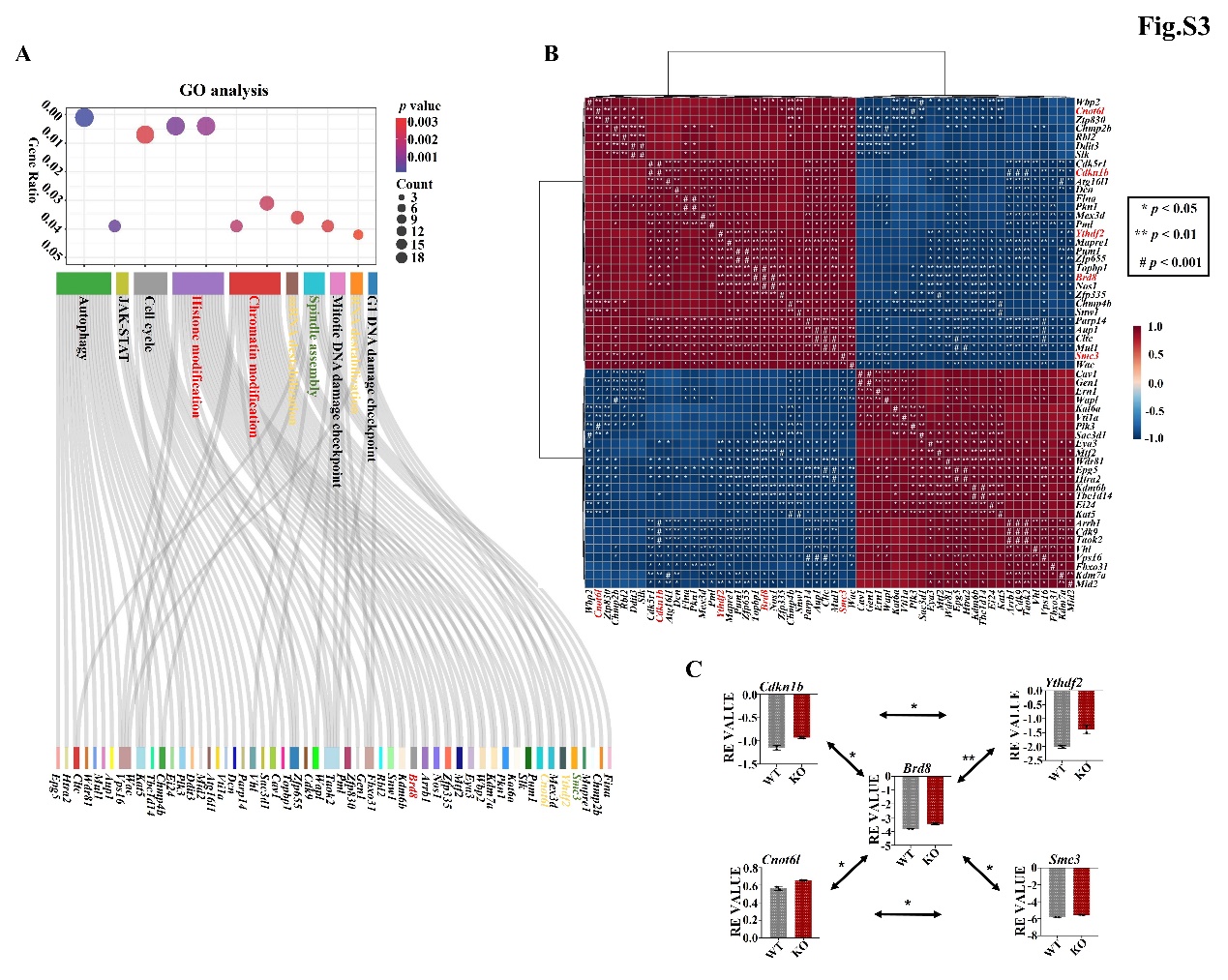


**Figure S3. The relevance of *Nsun5*^KO^-regulated alternative splicing genes**

**A**. GO analysis of the top 10 pathways for 391 differentiated genes with RE values at six months.

**B**. Fifty-six genes subjected to correlation analysis with statistics.

**C**. The RE value of *Brd8* was related to *Cdkn1b*, *Ythdf2*, *Cnot6l*, and *Smc3*, with significant differences between *Nsun5*^KO^ and WT mouse ovaries (**p*<0.05, ***p*<0.01).

**Abbreviation list:**

m5C: 5-methylcytosine

BSP-seq: Bisulfite genomic sequencing

Nsun5: NOP2/Sun RNA methyltransferase 5

MAD2L2: Mitotic arrest deficient 2 like 2

GDF9: Growth differentiation factor 9

BRD8: Bromodomain containing 8

PGCs: Primordial germ cells

GV: germinal vesicle

MII: metaphase II

MZT: Maternal-to-zygotic transition

USP19: ubiquitin specific peptidase 19

ANP32B: acidic nuclear phosphoprotein 32 family member B

GCLM: Glutamate-cysteine ligase modifier subunit

AS: alternative splicing

WT: Wild type

RED: relative expression difference

Co-IP: Coimmunoprecipitation

WB: Western blotting

**Table S1. Designations, sequences, and the sizes of real-time PCR amplicons**

| **Name** | **Sequence from 5'-3'** | **Size (bp)** |
| --- | --- | --- |
| GDF9 (H) Fw | AGCATAGTCTCCTTCGGGGT | 171 |
| GDF9 (H) Rev | TCCAAATGGTGGTAGAATTTGC |  |
| MAD2L2 (H) Fw | CAGACAAAGGAGGCAGACAAAG | 106 |
| MAD2L2 (H) Rev | GTGTGAGCGTGGTCATCCTT |  |
| GAPDH (H) Fw | GAAGGTCGGAGTCAACGGATTT | 223 |
| GAPDH (H) Rev | CTGGAAGATGGTGATGGGATTTC |  |
| BRD8 (M) Fw | GGAAGACTTGGATCTAGCGGAG | 136 |
| BRD8 (M) Rev | CACATCCAGCACTTCAGGGTGA |  |
| LAMB1 (M) Fw | GAACTACACGGTGAGGTTGGAG | 130 |
| LAMB1 (M) Rev | GCCAACAGTGAAGATGTCCAGC |  |
| SMC3 (M) Fw | CTTGTGTGGAAGTCACTGCTGG | 125 |
| SMC3 (M) Rev | AGGCAGGAAAGTCACCTCTCCA |  |
| PTGS1 (M) Fw | GAATGCCACCTTCATCCGAGAAG | 130 |
| PTGS1 (M) Rev | GCTCACATTGGAGAAGGACTCC |  |
| BRD8 (M-RT-PCR) Fw | AAGAGGATCAAGGAGAAGGCTATTT | 154 |
| BRD8 (M-RT-PCR) Rev | CTCTGTTGTACGAGAGTCCCCTA |  |
| GAPDH (M) Fw | TTCCAGTATGACTCTACCCACGGCA | 137 |
| GAPDH (M) Rev | GCACCAGCATCACCCCATTTG |  |

**H=Human; M=Mouse.**

**Table S2 KEY RESOURCES**

| **REAGENT or RESOURCE** | **SOURCE** | **IDENTIFIER** |
| --- | --- | --- |
| Rabbit anti-NSUN5 | Santa Cruz Biotechnology | Cat# sc-292513; RRID: AB_11151035 |
| Rabbit anti-MAD2L2 | ProteinTech | Cat# 12683-1-AP; RRID: AB_2139530 |
| Goat anti-GDF9 | R and D Systems | Cat# AF739; RRID: AB_2111517 |
| Rabbit anti-GCLM | ProteinTech | Cat# 14241-1-AP; RRID: AB_2107832 |
| Goat anti-NSUN5 | Santa Cruz Biotechnology | Cat# sc-104468; RRID: AB_2153930 |
| Mouse anti- alpha-Tubulin | Sigma | Cat# F2168; RRID: AB_476967 |
| Mouse Anti-H2A.X | Abcam | Cat# AB26350; RRID: AB_470861 |
| Alexa Fluor 488 anti-Mouse | ThermoFisher | Cat# A21202; RRID: AB_141607 |
| Alexa Fluor 555 anti-Rabbit | ThermoFisher | Cat# A31572; RRID: AB_162543 |
| **Chemicals, peptides and recombinant proteins** | | |
| M16 Media | Sigma | Cat# M7292 |
| M2 Media | Sigma | Cat# M7167 |
| Bovine Serum Albumin (BSA) | Sigma | Cat# B2064; CAS# 9048-46-8 |
| Triton X-100 | Sigma | Cat# 93443; CAS# 9036-19-5 |
| KSOM Mouse Embryo Media | Sigma | Cat# MR-020P |
| Milrinone | Sigma | Cat# M4659; CAS# 78415-72-2 |
| Mounting Medium With DAPI | Abcam | Cat# ab104139 |
| Pregnant Mare’s Serum Gonadotropin (PMSG) | Nanjing Aibei | Cat# M2620 |
| Mouse AMH ELISA kit | LSBio | Cat# LS-F6145-1 |
| Mouse E2 ELISA kit | MYBioSource | Cat# MBS8800210 |
| Mouse FSH ELISA kit | MYBioSource | Cat# MBS2700327 |
| **Experimental models: Cell lines** | | |
| KGN | RIKEN | Cat# RCB1154; RRID: CVCL_0375 |
| **Deposited data** |  |  |
| Raw data | This manuscript | PRJNA798568 |
| **Software and algorithms** |  |  |
| Image J | NIH | https://imagej.nih.gov/ij/ |
| GraphPad Prism | GraphPad Prism8 Software, La Jolla California USA | https://www.graphpad.com |
